# Use of Radiographic Criteria to Predict Outcomes Following Surgery for Pancreatic Cancer

**Published:** 2014-09-01

**Authors:** Rae Brana Reynolds, Justin Folloder

**Affiliations:** University of Texas MD Anderson Cancer Center, Houston, Texas

Surgical resection of the pancreas is the only treatment for pancreatic cancer that offers curative potential. Because other treatment modalities such as chemotherapy and radiation only offer palliation, surgical resectability is critical, as it dictates the treatment plan and ultimately serves as a determinant for long-term survival. Unfortunately, 80% of patients diagnosed with pancreatic cancer present with metastatic or locoregional disease at initial diagnosis (Chatterjee et al., 2012; Karmazanovsky, Fedorov, Kubyshkin, & Kotchatkov, 2005). These patients are therefore deemed ineligible for resection at initial diagnosis given that metastatic and locally advanced extrapancreatic disease are exclusion criteria for surgical treatment.

As such, pancreatic cancer has a grim prognosis, with an overall survival rate of only 6% (American Cancer Society, 2014). According to Hidalgo (2010), patients with earlier stages of disease deemed resectable have higher median survival rates (stage IA, 24.1 months; stage IB, 20.6 months; stage IIA, 15.4 months; stage IIB, 12.7 months) compared with patients with advanced stages of disease considered to be unresectable (stage III, 10.6 months; stage IV, 4.5 months). The implications for long-term survival accentuate the importance of identifying characteristics that distinguish resectable from unresectable disease at the outset of diagnosis and treatment planning.

The involvement of the superior mesenteric vein (SMV) or the portal vein (PV) by pancreatic cancer was historically considered a contraindication for surgical resection (Katz, Fleming, Pisters, Lee, & Evans, 2008). These vessels are adjacent to the pancreas and are at high risk for involvement by the pancreatic tumor.

There remains concern that resection and reconstruction of the involved SMV-PV during pancreatic cancer surgery is a high-risk procedure given the higher than usual risk for perioperative complications owing to the additional complexity of surgery. Additionally, it is thought that these patients are also at high risk for early systemic failure due to the advanced nature of the primary tumor, and at high risk for margin-positive resection with surgery alone. However, in centers where experienced surgical oncologists are performing a high volume of pancreatic cancer operations in a multidisciplinary setting, surgical outcomes have alleviated this concern (Evans et al., 2009). The quality of surgical resection affects long-term survival, and patients who undergo pancreatic cancer resection in high-volume hospitals with superior surgical oncologic expertise have higher survival rates than patients who undergo resection at low-volume centers, where surgical margins have been found to have a higher likelihood of being positive for residual disease (Bilimoria et al., 2008; Herman et al., 2008).

A 2012 study that examined hospital surgery volume, postoperative margin status, and long-term survival after pancreatic cancer resection found that high-volume hospitals were associated with significantly more cases of operative margins that were free from cancer (La Torre et al., 2012). The same study found that pancreatic resections at low-volume centers resulted in inferior operative margin outcomes and overall 5-year survival rate of patients (La Torre et al., 2012). In high-volume settings, there is more experience with safe and effective resection and reconstruction of the involved SMV and/or PV at the time of pancreatic cancer surgery, resulting in complete removal of the pancreatic tumor with R0/R1 outcomes. Hence, it is important to examine ways to develop standards in characterizing the tumor-vein interface (TVI) so that uniformity is established in discriminating between tumors that are potentially resectable or otherwise. The Table summarizes surgical margin clearance information referenced in this article.

**Table 1:**
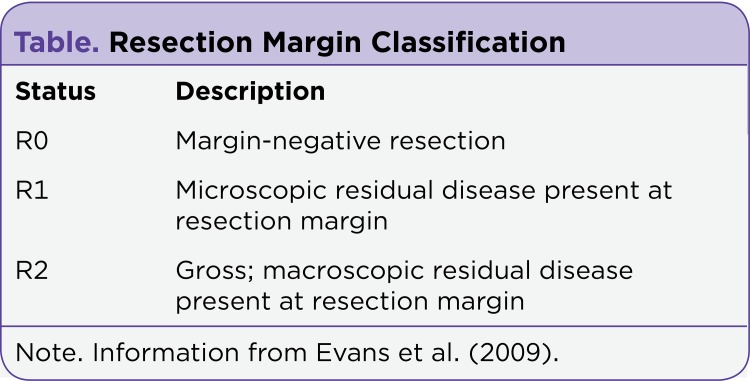
Resection Margin Classification

## Review of the 2014 tran cao et al. Article

Pancreatic cancer can present anywhere along the spectrum of resectable, borderline resectable, locally advanced, and unresectable disease. The article by Tran Cao et al. focuses on the borderline resectable category of tumors (Tran Cao et al., 2014). Borderline resectable disease has been categorized by the MD Anderson Cancer Center Pancreas Group according to three categories. Category A consists of disease that is questionable in terms of resection secondary to anatomic constraints. The level of vascular involvement is classified according to three conditions: tumor abutment ( 180°) of the SMA or celiac axis; tumor abutment or encasement (> 180°) of the circumference of the vessel of a short segment of the hepatic artery, usually at the origin of the gastroduodenal artery; short segment occlusion of the SMV, PV or SMV-PV confluence that is amenable to vascular resection and reconstruction because of patent venous access above and below the area of tumor-related occlusion. Category B centers on the concern for tumor biology, more specifically the notion of metastatic disease. Category C centers on each patient’s performance status and their tolerance for major abdominal surgery.

Within the borderline A cohort of patients, accurate classification of the extent of disease is critical in determining the eligibility for surgery. The goal of a complete margin-negative resection (R0) is significant to long-term survival, as several studies have suggested similar survival rates for margin- positive surgery when compared with survival rates for unresectable local-regional disease or locally advanced disease (Jemal et al., 2007; Alexakis et al., 2004; Sener, Fremgen, Menck, & Winchester, 1999).

The surgical oncology group at the University of Texas MD Anderson Cancer Center (MDACC)has established that in a highly select patient population, an R0 margin resection can be achieved through the addition of vein resection. It was done with low perioperative morbidity, and survival rates were comparable to those of patients who underwent an R0 resection without venous resection. Furthermore, it was achieved with a complication rate of 22%, a mortality rate of 1.6%, and a median survival of 22 months (Bold et al., 1999). Venous resection should be strongly considered if it yields a margin-negative resection (R0) since survival is improved when compared with a microscopic (R1) or macroscopic (R2) resection (Bold et al., 1999; Harrison & Brennan, 1998).

## Methods

This article summarized a study designed to assess the ability of radiographic criteria to predict the need for SMV-PV resection and the presence of histologic vein invasion. A system to categorize the TVI based on the pancreatic tumor’s relationship with the SMV-PV according to preoperative imaging was described, and its ability to accurately predict the need for venous resection and histologic vein involvement was evaluated. The study was conducted at the authors’ institution, MDACC, and examined all patients who underwent pancreaticoduodenectomy from 2004 to 2011 for pancreatic adenocarcinoma originating in the pancreatic head. Clinical data on these patients were retrieved from the pancreatic tumor database maintained by the institution’s pancreatic cancer surgery program within the Department of Surgical Oncology. Patients who did not undergo a preoperative multidetector computed tomography (CT scan) performed according to MDACC’s diagnostic imaging department pancreatic protocol within 3 months before pancreaticoduodenectomy were excluded from analysis.

Additionally, patients whose final surgical pathology report indicated pancreatic adenocarcinoma arising from an intraductal papillary mucinous neoplasm (IPMN) or mucinous cystic neoplasm (MCN) were excluded from the study as well. By these criteria, 11 patients who did not undergo preoperative CT scan per the institution’s pancreatic protocol within 3 months of pancreaticoduodenectomy, and 12 patients with final pathology demonstrating precursor IPMN or MCN lesions were excluded. Thus, 254 of 277 patients were eligible for the final analysis.

The pancreas protocol CT utilized consisted of images in two phases after injection of contrast, with positive or negative oral contrast as chosen by the protocoling radiologist. Bolus triggering was used to track the contrast in the vessels while it was injected into the patient to obtain images at the correct phases. Both phases were obtained at 2.5-mm slice thickness and reconstructed to 1.25- or 0.625-mm slice thickness. The images from the first phase were for analyzing the primary tumor, to identify variant arterial anatomy, and for assessing the relationship of the tumor to the arteries. The portal venous phase images were used to identify liver metastases and venous involvement. A dual-phase technique was used, as up to 40% of primary pancreatic tumors are of the same density of the pancreas and therefore essentially invisible (in the case of small operable cancers) on the later portal venous phase.

The preoperative pancreatic protocol CT scan of each eligible patient was reviewed by a single radiologist with expertise in gastrointestinal CT scan interpretation. The radiologist was blinded to each patient’s clinical history. The circumferential TVI between the primary pancreatic tumor and the SMV-PV was measured on axial CT scan images and categorized as (1) *none,* when there was no direct contact between the pancreatic tumor and the vessels, as they were separated by normal pancreas or a fat plane; (2) *TVI* *< 180*°, when the pancreatic tumor had interface with the vein at less than 180° of the vein’s circumference; (3) *TVI > 180*°, when there was interface of the pancreatic tumor with greater than 180° of the vessel’s circumference; or (4) *vascular occlusion,* when there was occlusion of the vessel as evidenced by the absence of contrast within the lumen of the vein in association with the adjacent pancreatic tumor.

The requirement for SMV-PV resection during pancreaticoduodenectomy was determined by the operating surgeon during surgery based on the interface between the pancreatic tumor and the vein. SMV-PV resection was performed when the primary tumor was adherent to and not easily separated from the vein. Reconstruction of the vein was completed with primary closure or patch venoplasty when tangential resection was performed, whereas bypass graft with autologous internal jugular vein or bioprosthetic material was performed for segmental vein resection. The surgical specimen was then submitted for review, and the closest distance between the cancer cells and the margin where the superior mesenteric artery (SMA) was resected was measured microscopically. When applicable, the resected portion or segment of the SMV-PV was also submitted for review, evaluated for tumor involvement, and subsequently categorized as (1) no tumor involvement, (2) tumor invasion into the tunica adventitia, (3) tumor invasion into the tunica media or intima, or (4) tumor invasion into the SMV-PV lumen with or without thrombus. Patients who did not require or undergo SMV-PV resection during pancreaticoduodenectomy were considered to have no tumor involvement of the vein.

In addition to evaluating the ability of radiographic criteria to predict the need for venous resection, the presence of histologic invasion of the vein wall by tumor and the overall survival of patients who had and had not received chemoradiation prior to pancreaticoduodenectomy were also examined. In this study, patients treated with preoperative chemoradiation received three-dimensional, conformal radiation therapy of 30 Gy in 10 fractions or 50 Gy in 28 fractions with concomitant capecitabine, 5-fluorouracil, or gemcitabine. In some cases, gemcitabine-based chemotherapy was delivered prior to chemoradiation and subsequent pancreaticoduodenectomy.

## Analysis and Results

The investigators employed t-test and Pearson chi-squared analysis to assess differences in the clinical characteristics and demographics by TVI categories, venous resection, and final pathology results. Assessment of areas under the curve (AUC) was performed by constructing receiver operating characteristic (ROC) curves. These curves allowed the investigators to assess the ability of the TVI classification to accurately predict the intraoperative need for vein resection and the presence of vein invasion by tumor. Survival analysis was performed via the Kaplan-Meier method to estimate median survival for each clinical and demographic factor, and Cox regression analysis was used to identify hazard ratios for the variables under examination.

The CT scan review by the radiologist revealed that of the 254 patients eligible for review, the TVI breakdown consisted of 62 patients (24.4%) with no TVI, 154 patients (60.6%) with TVI 180°, 28 patients (11%) with TVI > 180°, and 10 patients (3.9%) with venous occlusion associated with the pancreatic tumor. SMV-PV resection was performed in 8 of the 62 patients (12.9%) who did not have TVI, 56 of the 154 (36.4%) with TVI 180°, 25 of the 28 (89%) with TVI > 180°, and 9 of the 10 (90%) with venous occlusion (*p* < .001). The rate of microscopically negative margins (R0 resection) was similar across TVI groups (*p* = .25), and microscopically negative margins were achieved in over 90% of resected cases, regardless of the preoperative TVI category.

Chemoradiation with or without induction chemotherapy was delivered to 194 patients (76.4%) prior to pancreaticoduodenectomy during which SMV-PV resection and reconstruction was performed in 98 patients (38.6%). Among these 98 patients, 93 had complete histopathologic assessment of the vein. The vein wall was invaded by cancer in 64 patients (68.8%), with 17 having involvement of the tunica adventitia, 42 having involvement of the tunica media/intima, and 5 having invaded the vein lumen. The investigators reported the clinical characteristics and outcomes for each TVI category and noted that primary cancers with greater circumferential TVI were larger, more likely to have been treated with preoperative chemoradiation, and more likely to have required resection of the SMV-PV during pancreaticoduodenectomy (*p* < .001).

An ROC curve was constructed to evaluate the ability of the preoperative TVI system to accurately predict whether SMV-PV resection would be necessary. The AUC of the ROC curve indicates how the TVI system can preoperatively discriminate between patients who require SMV-PV resection during pancreatic cancer extirpation and those who do not. The results showed an AUC of 0.734. An ROC curve was also constructed to evaluate the ability of the TVI system to accurately predict SMV-PV tumor invasion; this was calculated at 0.768. It represents the ability of the TVI system to discriminate preoperatively between patients whose postoperative histopathology will reveal tumor involvement of the SMV-PV and those whose will not. Given these results, the authors stated that the TVI achieved fair accuracy in predicting the need for venous resection and histopathologic vein invasion.

As for long-term outcomes, the investigators evaluated progression-free survival (PFS) and overall survival (OS). Pancreatic cancer surgery with SMV-PV resection and reconstruction was associated with a shorter median PFS (16.1 vs. 19.6 months; *p* = .013) and OS (27.8 vs. 44.5 months; *p* = .002) compared with surgery without SMV-PV resection. Additionally, the investigators also found that tumor vein involvement confirmed by histology was associated with a shorter median PFS (15.6 vs. 19.6 months; *p* = .001) and OS (27 vs. 40.4 months; *p* = .001) compared with absence of tumor vein involvement. Similarly, patients with a TVI > 180° had a shorter median PFS (15.9 vs 18.2 months; *p* = .006) and OS (30.9 vs. 37.3 months; *p* = .030) than patients with TVI < 180°. Of note, patients who had no TVI were grouped with those who had a TVI < 180°, as the authors pointed out that the survival curves of these patients were similar.

## Limitations and Strengths

The overall study has some limitations in that only the primary preoperative scan was reviewed, and the changes in TVI in response to preoperative neoadjuvant treatment were not examined. The authors acknowledged that it would have been useful to assess cases and note where tumor downstaging occurred. The study also excluded patients whose planned resections were aborted due to intraoperative findings.

Despite these acknowledged limitations, the authors pointed out the study’s strengths, such as having one radiologist review the preoperative CT images for increased integrity in the uniform use of the TVI characterization system. All patients were also evaluated via a standardized, high-quality protocol specifically designed for pancreatic evaluation. In addition, the indications for surgical resection were also standardized, as were the surgical techniques and histopathologic evaluation of surgical specimens. The fact that evaluation and treatment occurred in a high-volume center experienced in the care of patients with borderline resectable disease was also noted as a fundamental strength of the study.

## Practical Implications

Accurately characterizing pancreatic tumors is fundamental and critical for overall treatment planning. Concurrent vascular resection and reconstruction at the time of major pancreatic surgery has been, and continues to be, controversial due to the complexity of the surgical procedure itself, the limited experience of many surgeons regarding the described vascular work, and the potential for synergistic perioperative morbidity and mortality. Moreover, most pancreatic surgeons desire to minimize this perioperative risk given the aggressive nature of pancreatic adenocarcinoma, which has a known poor survival rate. Over the past 20 years, advances in pancreatic surgery have made it possible to resect and reconstruct the SMV-PV as well as the hepatic artery. This operation can be done safely and effectively as part of a pancreatectomy, when offered to the appropriate, well-selected patient population. The end result provides the potential for improved survival for such surgical patients.

Additionally, a multidisciplinary approach utilizing neoadjuvant chemotherapy and chemoradiation can facilitate the selection of patients whose tumors present favorable biology for such major operations. This neoadjuvant pathway is especially useful for localized tumors that are technically resectable but are at increased risk for R1 or R2 resection secondary to their close anatomic relationship to major vascular structures (Katz, Ahmad, & Nelson, 2013). Achieving a margin-negative (R0) resection is fundamental for the potential cure; the definition of borderline resectable pancreatic cancer and the proximity and involvement of major vascular structures often necessitates a combined resection and reconstruction of the SMV-PV region, in the absence of distant disease or disease involving structures that would preclude surgery (the SMA, celiac axis, and often the common hepatic artery; Kang, Hwang, Choi, & Lee, 2013). As previously outlined, the role for aggressive vascular resection and reconstruction is a critical aspect to facilitate complete tumor clearance, achieving an R0 resection for borderline resectable tumors.

The ability to standardize a preoperative classification system for describing TVI based on preoperative radiography is critical in determining resectability and in planning for pancreatic cancer surgery. It also has the potential to predict histopathologic information, with practical implications for assessing long-term survival. The system proposed by the authors is simple and does away with ambiguous terminology.

Although there is no widely accepted standard for classification of tumor at this time, it is valuable that experts are evaluating ways to establish uniformity to enhance the evaluation, treatment, and ongoing research efforts that benefit patients with pancreatic cancer.
